# Active Targeting Hyaluronan Conjugated Nanoprobe for Magnetic Particle Imaging and Near-Infrared Fluorescence Imaging of Breast Cancer and Lung Metastasis

**DOI:** 10.1021/acsami.4c01623

**Published:** 2024-05-17

**Authors:** Chia-Wei Yang, Kunli Liu, Cheng-You Yao, Bo Li, Aniwat Juhong, A. K. M. Atique Ullah, Harvey Bumpers, Zhen Qiu, Xuefei Huang

**Affiliations:** † Department of Chemistry, Michigan State University, East Lansing, Michigan 48824, United States; ‡ Institute for Quantitative Health Science and Engineering, Michigan State University, East Lansing, Michigan 48824, United States; § Department of Electrical and Computer Engineering, Michigan State University, East Lansing, Michigan 48824, United States; ∥ Department of Surgery, Michigan State University, East Lansing, Michigan 48824, United States; ⊥ Department of Biomedical Engineering, Michigan State University, East Lansing, Michigan 48824, United States

**Keywords:** breast cancer, fluorescence imaging, hyaluronan, magnetic particle imaging, multimodality imaging

## Abstract

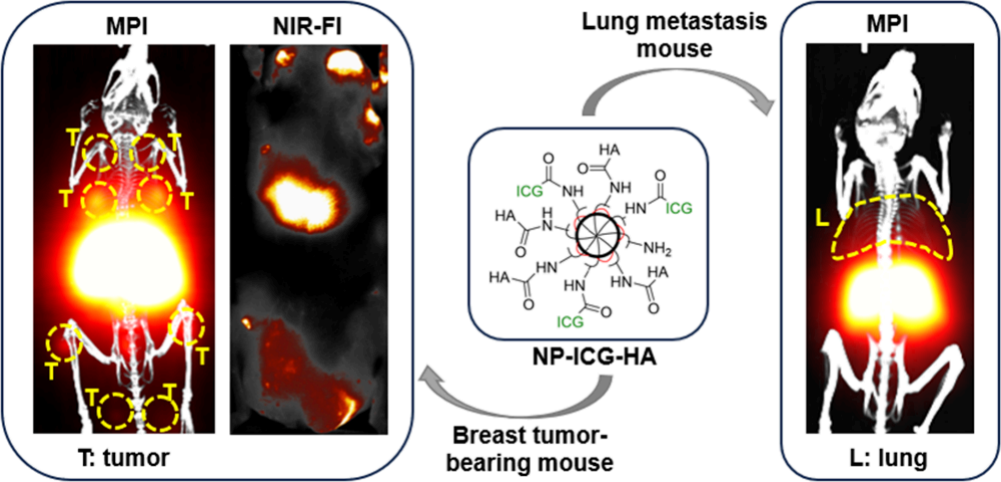

A major contributing cause to breast cancer related death is metastasis. Moreover, breast cancer metastasis often shows little symptoms until a large area of the organs is occupied by metastatic cancer cells. Breast cancer multimodal imaging is attractive since it integrates advantages from several modalities, enabling more accurate cancer detection. Glycoprotein CD44 is overexpressed on most breast cancer cells and is the primary cell surface receptor for hyaluronan (HA). To facilitate breast cancer diagnosis, we report an indocyanine green (ICG) and HA conjugated iron oxide nanoparticle (NP-ICG-HA), which enabled active targeting to breast cancer by HA-CD44 interaction and detected metastasis with magnetic particle imaging (MPI) and near-infrared fluorescence imaging (NIR-FI). When evaluated in a transgenic breast cancer mouse model, NP-ICG-HA enabled the detection of multiple breast tumors in MPI and NIR-FI, providing more comprehensive images and a diagnosis of breast cancer. Furthermore, NP-ICG-HAs were evaluated in a lung metastasis model. Upon NP-ICG-HA administration, MPI showed clear signals in the lungs, indicating the tumor sites. This is the first time that HA-based NPs have enabled MPI of cancer. NP-ICG-HAs are an attractive platform for noninvasive detection of primary breast cancer and lung metastasis.

## 1 Introduction

Breast cancer is the most diagnosed cancer type in women, with more than 2.3 million new cases in 2020 worldwide. Through the advances in early detection and treatment strategies, the survival rate of breast cancer patients has been significantly improved.^1,2^ On the other hand, breast cancer metastasis remains a major mortality factor. The locations of metastasis are often challenging to detect. While the lung, liver, and bone are common sites for breast cancer metastasis, they often show little symptoms until large areas of the organs have been occupied by metastatic cancer cells. Thus, it is critical that methods are available to detect primary and metastatic breast cancer.^3−5^

Noninvasive cancer imaging is an emerging field. Various scanning modalities have been utilized for cancer imaging in the lung including computed tomography (CT),^6^ positron emission tomography (PET),^7−9^ magnetic resonance imaging (MRI),^10,11^ and optical imaging.^12^ To better determine the presence of lung metastasis, multimodal imaging has been applied. ^64^Cu-labeled interleukin 18 or a ^89^Zr-labeled monoclonal antibody enables breast cancer lung metastasis imaging and evaluation with PET and CT/optical imaging.^13,14^ However, PET and CT use ionizing radiation, which may be harmful to patients.^15^

Nanoparticle-based contrast agents can significantly aid in cancer imaging. Nanoworms (NWs) bearing indocyanine green (ICG) (NW-ICGs) have been applied to primary breast cancer detection in mice.^16,17^ The accumulation of the nanoprobes in cancer was presumably due to the passive targeting of NWs through the enhanced permeability retention (EPR) effect.^18−20^ However, the breast cancer lung metastasis typically has small tumor masses compared to the primary tumor; thus, a less prominent EPR effect is expected.^21,22^ To enhance NP accumulation in metastatic sites, cell surface receptors on tumor cells may be targeted. CD44 is a glycoprotein that is overexpressed on various types of cancerous cells, including breast cancer.^23,24^ CD44 is known to play a key role in metastasis and relapse of breast cancer.^25,26^ Hyaluronan (HA) is the major endogenous ligand of CD44.^23,27−29^ Herein, we report an ICG and HA conjugated iron oxide nanoparticle (NP-ICG-HA), which can bind well with CD44. This can enable active NP targeting metastatic breast cancer cells in the lungs and its detection with magnetic particle imaging (MPI) and near-infrared fluorescence imaging (NIR-FI).

MPI is an attractive imaging modality that detects superparamagnetic materials and does not require ionizing radiation. Compared to magnetic resonance imaging (MRI), MPI has multiple advantages such as high sensitivity, high imaging contrast with nearly no background, and the possibility for quantification of tracers while maintaining the high depth for imaging.^30−32^ Fluorescence imaging can complement the MPI results with the advantage of relatively high resolution. ICG is an NIR fluorescence dye approved by the Food and Drug Administration (FDA) as a clinical imaging agent.^33−39^ NIR fluorescence dyes have their emission windows at 700–900 nm, enabling deeper tissue penetration when used in animal study. Thus, it could provide stronger signals compared to fluorescence dyes with emission maxima in the visible region. While ICG-HA NPs have been reported previously,^40,41^ they have not been tested for MPI-based cancer and metastasis imaging.

## 2 Results

### Synthesis and Characterization of NP-ICG-HA

**Scheme 1:**
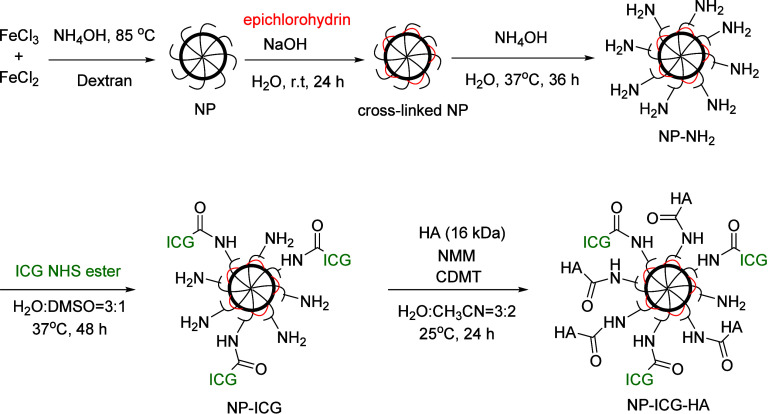
Synthesis of NP-ICG-HA

The iron oxide nanoparticles (NPs) were synthesized by the modified coprecipitation method from Fe(III) and Fe(II) salts in the presence of dextran.^42^ The dextran on the surface of NPs was then cross-linked with epichlorohydrin, followed by introduction of amine moieties by reacting with ammonium hydroxide to form aminated NP (NP-NH_2_). ICG was installed onto NP-NH_2_ with an ICG-*N*-hydroxysuccinimide (NHS) ester producing NP-ICG. HA was then conjugated to NP-ICG through amide bond formation, leading to NP-ICG-HA (Scheme 1).^43−45^

**Figure 1:**
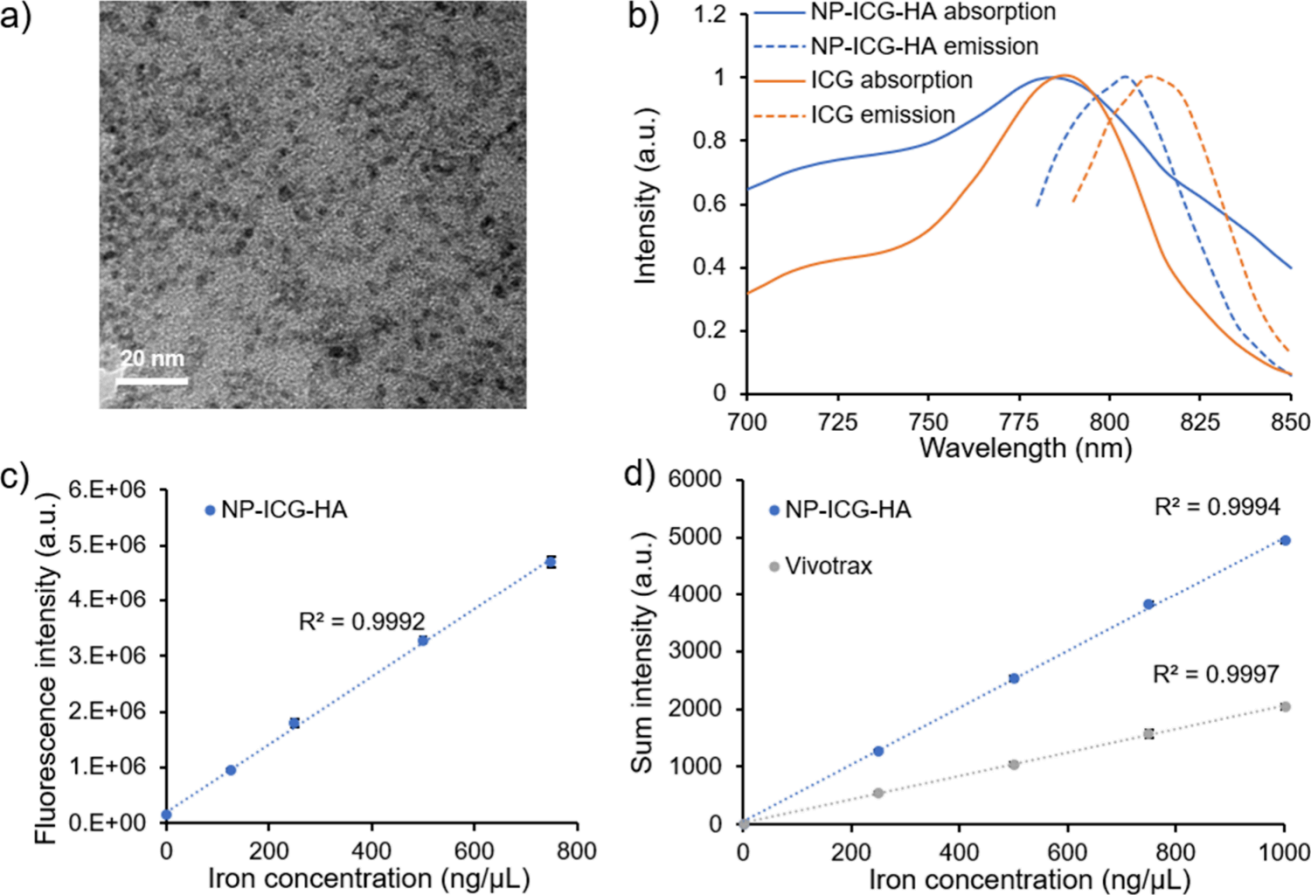
(a) TEM image of NP-ICG-HA. (b) Normalized UV–vis absorption and emission spectra of NP-ICG-HA and free ICG. Excitation: 730 nm. (c) Integrated fluorescence signal intensity vs iron concentration of NP-ICG-HA (*n* = 3). (d) MPI signal intensities vs iron concentration for NP-ICG-HA and VivoTrax (*n* = 3).

The nanomaterials were characterized first through dynamic light scattering (DLS). The average hydrodynamic diameters of NP, NP-ICG, and NP-ICG-HA were 67, 122, and 208 nm, respectively, indicating the successful conjugations of ICG and HA to the NP (Figure S1a). The zeta potential values of the particles were also measured (Figure S1b). Aminated NP had a positive zeta potential (22.3 mV) likely due to the presence of ammonium ions on the surface. Upon conjugation of NP with ICG, the zeta potential of the resulting NP-ICG became less positive (16.9 mV) as some of the amine moieties were amidated. The immobilization of HA to NP-ICG further decreased the zeta potential to negative values (−14.2 mV) presumably due to the negatively charged nature of HA at neutral pH. The morphology of NP-ICG-HA was analyzed by transmission electron microscopy (TEM) (Figure 1a), which showed that the average diameter of the iron oxide core was ∼3 nm. The significantly larger sizes observed by dynamic light scattering compared to TEM were presumably due to the polysaccharides attached on the NP surface, which were not readily visible under TEM. When comparing the TEM images of NP, NP-ICG, and NP-ICG-HA (Figure S2a), no significant differences were observed in size, as they were made of the same core. Elemental analysis for NP-ICG-HA was conducted by energy dispersive X-ray spectroscopy (EDX) (Figure S2b), which indicated the presence of iron, carbon, and oxygen elements in the particles. The fluorescence spectra of NP-ICG-HA were also acquired. Both the absorption and emission maximum peaks of NP-ICG-HA were blue-shifted by 5 nm as compared to those of the parent molecule ICG (Figure 1b). The blue-shifts of the absorption and emission maxima could be due to the metal-enhanced fluorescence effect^46^ or the nanoaggregation of ICG^47,48^ when conjugated with NPs. The intensities of fluorescence signals of NP-ICG-HA were linear with the nanoparticle iron concentration (Figure 1c). The iron concentration of NP-ICG-HA was determined by inductively coupled plasma optical emission spectrometry (ICP-OES). The MPI signal strength of NP-ICG-HA was linearly correlated to the nanoparticle iron concentration (Figure 1d). The performance of NP-ICG-HA in MPI was evaluated by benchmarking against the commercially available carboxydextran-coated superparamagnetic iron oxide nanoparticle (SPION): VivoTrax. The NP-ICG-HA was more sensitive than VivoTrax, giving stronger MPI signals at the same level of iron. The potential magnetic hysteresis of the nanoparticles was measured by the superconducting quantum interference device (SQUID) magnetometer at 300 K (Figure S3). No hysteresis loops were observed by SQUID, indicating the superparamagnetic property of NP-ICG-HA. The masses of iron in NP-ICG-HA and VivoTrax were determined by ICP-OES. The saturation magnetization of NP-ICG-HA (93.0 emu/g) is 2 times higher than that of VivoTrax (42.7 emu/g), explaining the higher sensitivity of NP-ICG-HA in MPI.

To ascertain HA immobilized on the NP-ICG-HA can bind with CD44, a competitive enzyme-linked immunosorbent assay (ELISA) was set up to measure the abilities of NP-ICG-HA to compete with HA for CD44 binding.^49^ As shown in Figure S4, NP-ICG-HA significantly reduced the level of binding of HA with CD44. In contrast, NP-ICG did not have much effect on HA/CD44 binding, confirming the important roles of HA in NP-ICG-HA/CD44 interactions (Figure S4). To evaluate the biocompatibility of NP-ICG-HA, cell viability assays were performed with RAW 264.7 cells (Figure S5). No major changes in cell viability were recorded when the cells were incubated with various concentrations (0.13–0.5 mg of Fe/mL) of NP-ICG-HA, demonstrating the material has no significant toxicity to cells at the concentrations evaluated.

### Binding of NP-ICG-HA with CD44-Expressing Breast Cancer Cells *In Vitro*

**Figure 2:**
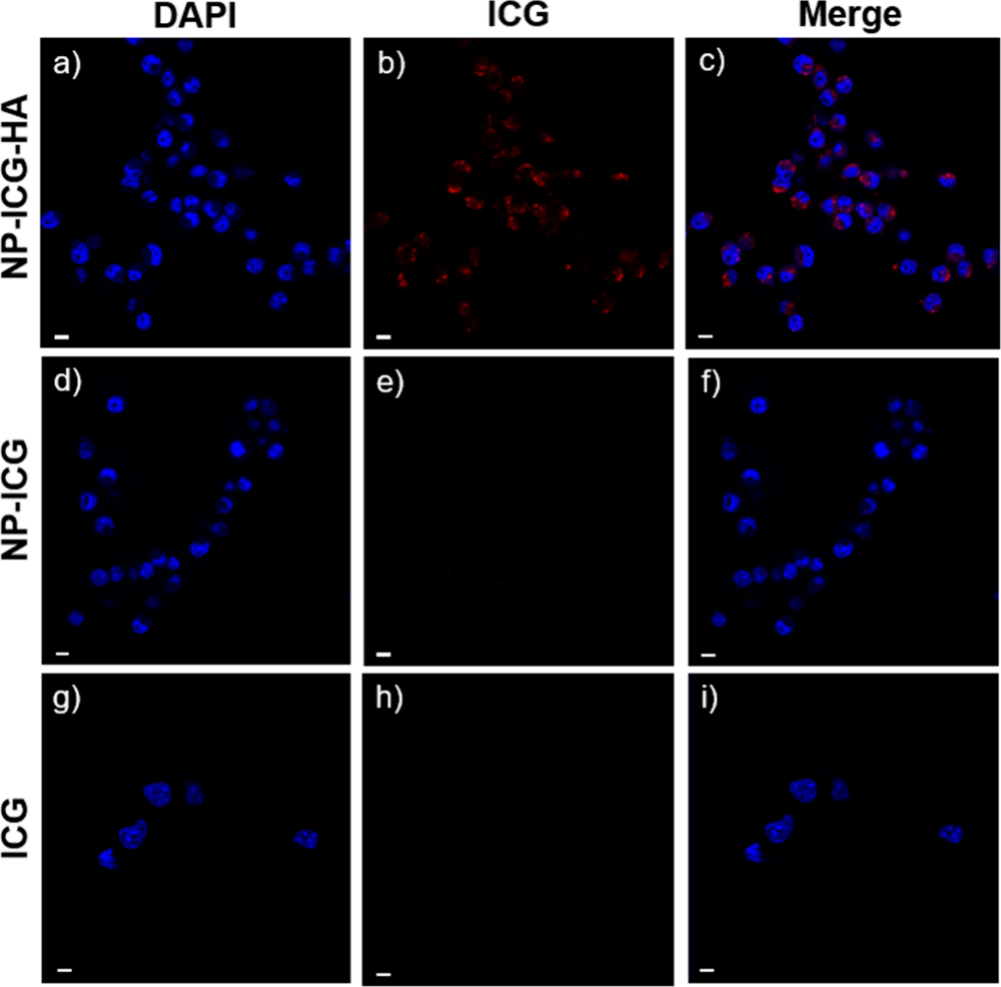
Fluorescence images measured by confocal microscopy. 4T1 cells were incubated with NP-ICG-HA and then DAPI, followed by washing with PBS three times after each staining, and imaged (a) DAPI channel, (b) ICG channel, and (c) merge of both. 4T1 cells were incubated with NP-ICG and then DAPI, followed by washing with PBS three times after each staining as the control group and imaged (d) DAPI channel, (e) ICG channel, and (f) merge of both. 4T1 cells were incubated with ICG and then followed by the same washing procedure and imaged (g) DAPI channel, (h) ICG channel, and (i) merge of both. Scale bars are 10 μm.

To examine the ability of NP-ICG-HA to detect cancer cells, CD44 expressing 4T1 cells^50^ were incubated with NP-ICG-HA, NP-ICG, or ICG at the same ICG fluorescence intensities for 2 h at 37 °C followed by thorough washing with PBS to remove the unbonded particles. Cells were then imaged by confocal microscopy (Figure 2). The images showed much stronger ICG signals in cells incubated with NP-ICG-HA, suggesting that NP-ICG-HAs were taken up more by the cells as compared to NP-ICG and ICG. The incubated cells were further stained with Prussian blue to detect iron present intracellularly (Figure S6). Cells incubated with NP-ICG-HA exhibited much stronger blue staining than those treated with NP-ICG, supporting the idea that more NP-ICG-HA particles were present in 4T1 cells.

### NP-ICG-HA Enabled Multimodality Imaging of Breast Cancer in the MMTV-PyMT Spontaneous Cancer Model

To evaluate the cancer imaging ability of NP-ICG-HA, the MMTV-PyMT transgenic mouse model is established.^51,52^ MMTV-PyMT mice spontaneously develop palpable mammary tumors in 4–6 months, which can mimic human breast cancer with a native microenvironment. The breast tumor tissues dissected from MMTV-PyMT mouse were first subjected to CD44 immunohistochemistry (IHC) staining confirming the expression of CD44 in these tissues (Figure S7).

**Figure 3:**
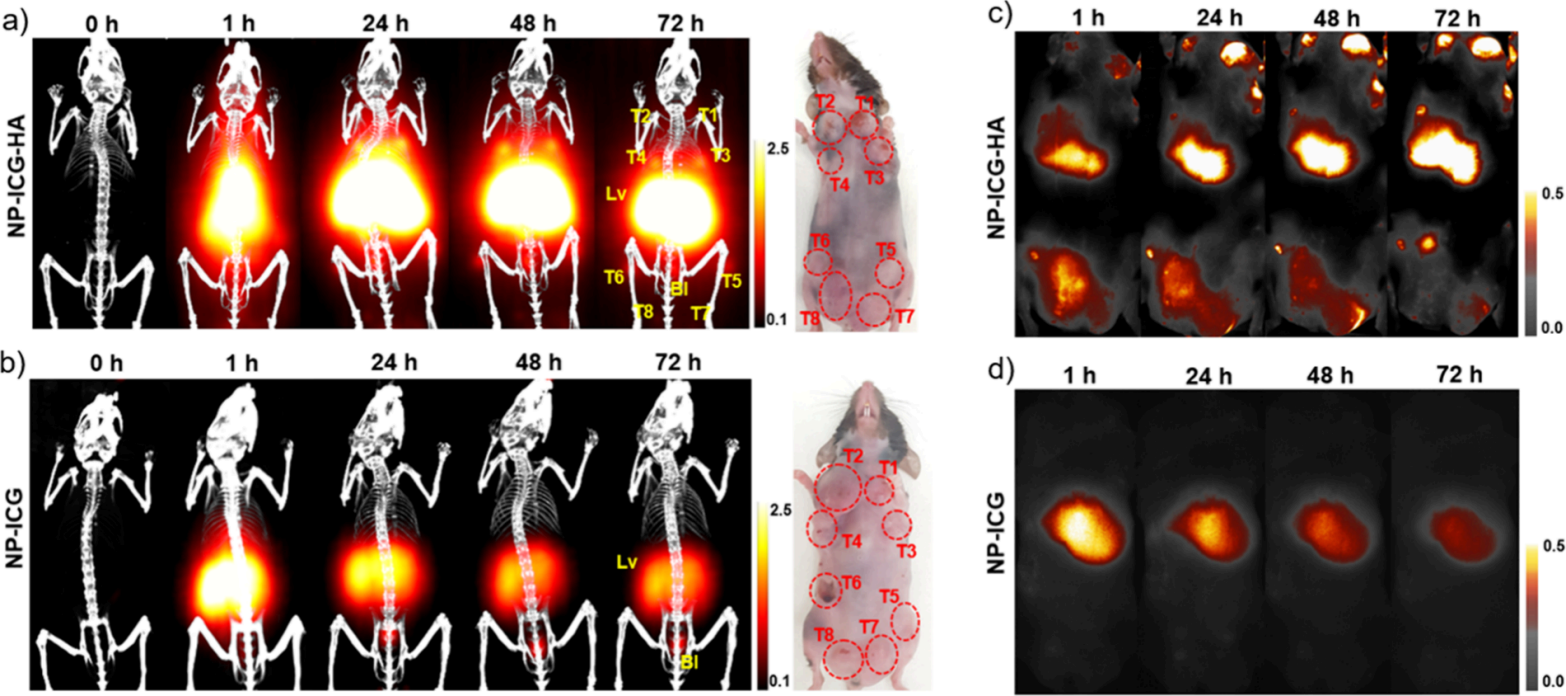
Imaging of MMTV-PyMT mice bearing multiple mammary tumors (*n* = 4 for each group). (a) 3D MPI images at indicated time points co-registered with CT skeletal scan images of mice injected with NP-ICG-HA. (b) 3D MPI images at indicated time points co-registered with CT skeletal scan images of mice injected with NP-ICG as a control group. (c) NIR-FI images of mice injected with NP-ICG-HA at various time points. (d) NIR-FI images of mice injected with NP-ICG as a control group at various time points.

Five-month-old female MMTV-PyMT mice (*n* = 4) were administered with NP-ICG-HA (8 mg of iron/kg of body weight) through the tail vein. These mice were then imaged with MPI and fluorescence over 72 h (Figure 3a). NP-ICG (8 mg of iron/kg of body weight) without conjugated HA was injected to another batch (*n* = 4) of MMTV-PyMT mice as the control group (Figure 3b). 3D MPI images were co-registered with CT images to provide anatomical information (Figure 3). In the NP-ICG-HA group, preinjection scanning showed no MPI signals, indicating low endogenous iron concentration. In comparison, images acquired 1 h postinjection showed MPI signals all over the mouse body, suggesting NP-ICG-HA was in the vasculature system. Twenty-four hours postinjection, strong MPI signals were observed in liver area as well as in the tumor area, showing NP-ICG-HA can accumulate in breast tumors. In contrast, for control mice receiving NP-ICG, particles were taken up by the liver and stayed in the liver for a shorter period. Moreover, no significant MPI signals were found in the tumor areas over the 72 h period in these mice. To confirm the MPI results, fluorescence imaging was performed, enabled by the ICG attached on NPs. Consistent with MPI studies, in the NP-ICG-HA group, fluorescence images showed significant signals in liver and tumor areas (Figure 3c), with no fluorescence in tumor areas in mice administered with NP-ICG (Figure 3d).

To further confirm the role of HA in targeting NP-ICG-HA to breast tumors, a mixture of NP-ICG-HA and free HA was injected intravenously into tumor-bearing MMTV-PyMT mice. The free HA could bind with the HA receptor (CD44) in breast tumors, which could competitively inhibit the binding between NP-ICG-HA and CD44 in tumors. Compared to mice receiving NP-ICG-HA, the MPI and fluorescence images showed significantly lower signals in tumor areas in the presence of free HA (Figures S8a and S8c). The signals in the tumor area were integrated and plotted for both the NP-ICG-HA group and the NP-ICG-HA + free HA group in MPI and NIR-FI results at 24 h postinjection (Figures S8b and S8d). A 95% reduction of signals in the MPI and an 88% reduction of signals in the fluorescence images were observed from the NP-ICG-HA group to the NP-ICG-HA + free HA group, respectively, indicating that tissue accumulation of NP-ICG-HA *in vivo* was HA dependent.

### Confirmation of MMTV-PyMT Mouse Imaging via *Ex Vivo* Analysis of the Tissues

**Figure 4:**
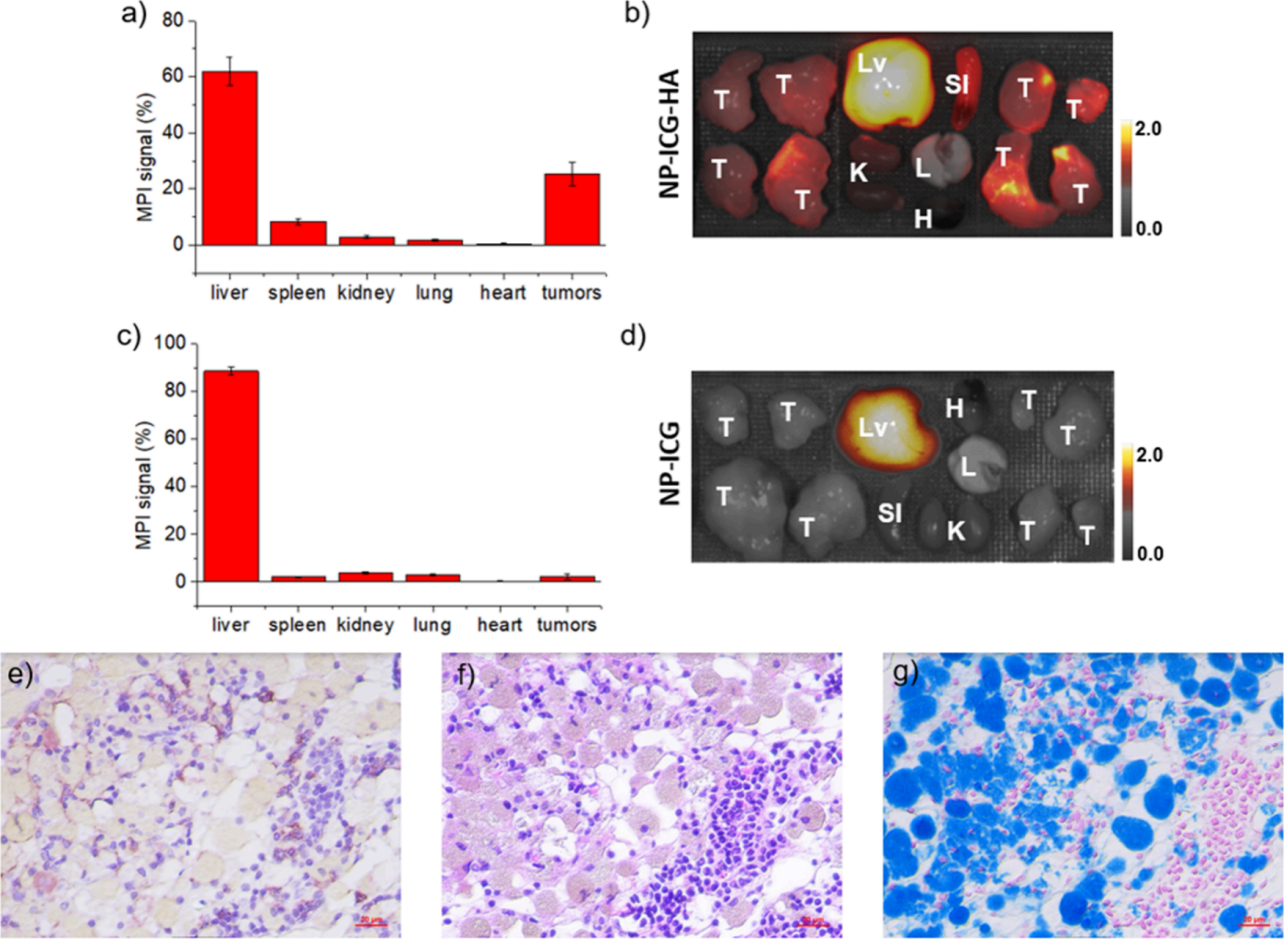
MMTV-PyMT mice (*n* = 4 for each group) bearing multiple mammary tumors injected with NP-ICG-HA or NP-ICG and then sacrificed at 72 h postinjection. (a) Percentage of MPI signals measured *ex vivo* in main organs and (b) *ex vivo* NIR-FI images of NP-ICG-HA group. (c) Percentage of MPI signals measured *ex vivo* and (d) *ex vivo* NIR-FI images of the NP-ICG control group. T: tumor; Lv: liver; K: kidney; Sl: spleen; H: heart; and L: lung. Histological analysis of tumor tissues from MMTV-PyMT mice receiving NP-ICG-HA. (e) Anti-CD44 IHC stain, (f) H&E stain, and (g) Prussian blue (iron showed blue) followed by nuclear fast red counterstain.

To confirm the *in vivo* imaging results, mice were euthanized 72 h postinjection, and the mouse organs were extracted and imaged by MPI and NIR-FI. The biodistribution of NP-ICG-HA was examined through quantification of MPI images of the organs acquired (Figure 4a). Significantly higher MPI signals (25.2% of the total signals from all organs extracted) were found in excised tumors. The *ex vivo* fluorescence signals (Figure 4b) corroborated the MPI results. Biodistribution of nanoparticles in the control group of mice receiving NP-ICG was performed parallelly. Signal quantification showed only strong MPI/fluorescence signals from excised liver, with little signals from the tumor (Figures 4c and 4d). To further confirm the accumulation of NP-ICG-HA in tumors, histopathological analysis was conducted in excised tissues. The CD44 expression of excised tissues were examined by immunohistostaining (Figure 4e) with the adjacent slides of tissues stained with hematoxylin and eosin (H&E) and Prussian blue (Figures 4f and 4g). The dense nucleus stain area in H&E slide was co-localized with brownish area in the CD44 IHC slide, indicating the CD44 expression in tumors. The extensive blue color observed in Prussian blue staining confirmed the presence of NP-ICG-HA in tumors.

### NP-ICG-HA Enabled Multimodality Imaging of Breast Cancer Lung Metastasis

To test the ability of NP-ICG-HA to detect cancer metastasis, a breast cancer lung metastasis mouse model was built by injecting 4T1-Luc2 breast cancer cells into female BALB/c mice through the tail vein. Bioluminescence imaging showed that 4T1-Luc2 cells were accumulated in the lung areas of mice, which could mimic metastasis of breast cancer to the lung.

**Figure 5:**
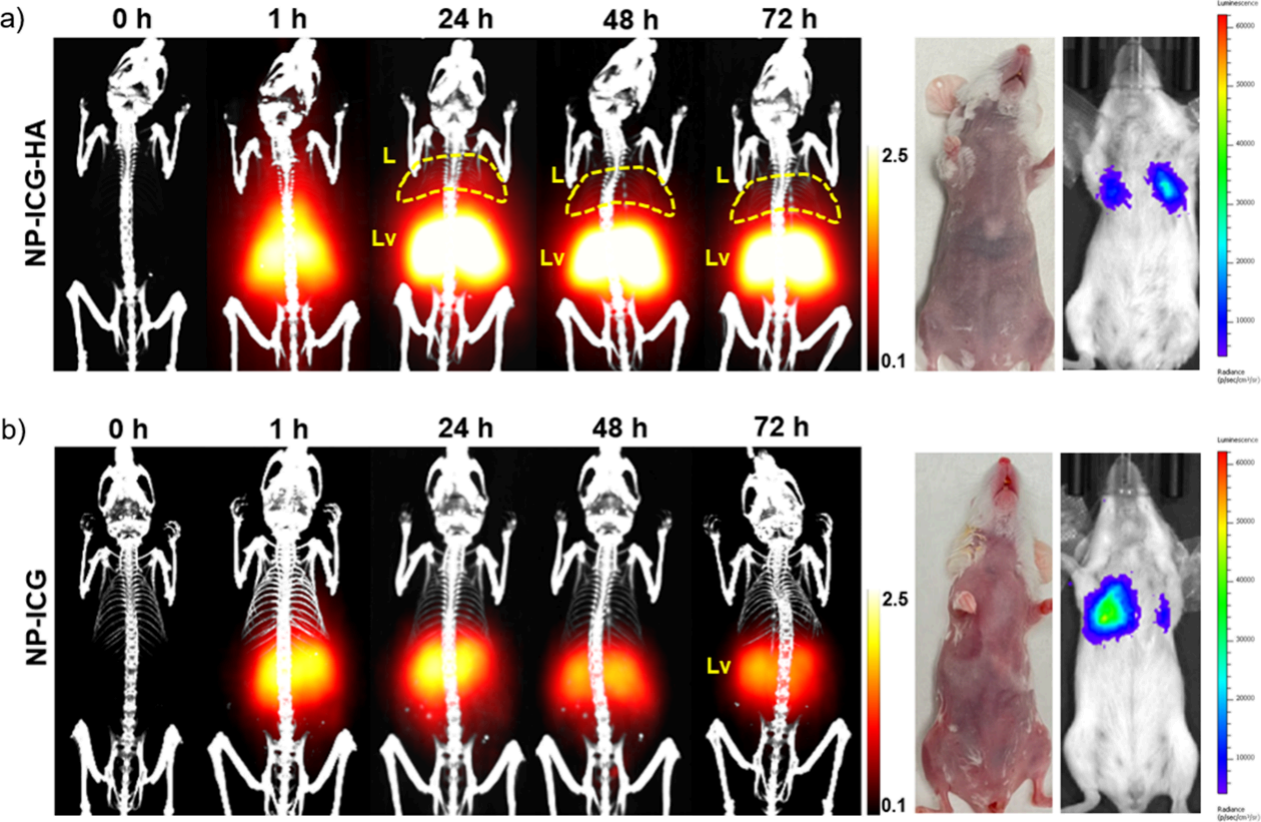
Imaging of 4T1 lung metastasis mice (*n* = 4 for each group). (a) 3D MPI images at indicated time points co-registered with CT skeletal scan images (left) of mice injected with NP-ICG-HA. The photo (middle) of the mouse and bioluminescence imaging (right) measured by IVIS. (b) 3D MPI images at indicated time points co-registered with a CT skeletal scan images of mice injected with NP-ICG as a control group.

NP-ICG-HA or NP-ICG was injected at the same iron dose (8 mg of iron/kg of body weight) through the tail vein to mice with 4T1 cancer cells in the lung, which was followed by MPI and NIR-FI over 72 h. In the NP-ICG-HA group, MPI images showed strong signals in abdominal and chest areas at 1 h postinjection, indicating the particles were still in the circulation (Figure 5a). At 24 h, strong signals were observed in the liver area, and signals were found in both left and right lungs. However, in the NP-ICG control group, MPI signals were only visible in the liver area at all time points (Figure 5b). To exclude the possibility that NP-ICG-HA particles nonspecifically accumulated in the lungs, BALB/c mice without tumor were administered with NP-ICG-HA. No MPI signals were found in the lung areas of these mice (Figure S9). The results supported that NP-ICG-HA selectively accumulated in the lungs of 4T1 lung metastasis mice. To confirm the MPI results, fluorescence signals were measured at the corresponding time points (Figure S10). However, no significant signals were found in lung areas, which could be because the fluorescence signals were too weak to penetrate through the chest to be detected *in vivo*. In this *in vivo* data set, MPI showed its superiority to fluorescence imaging when detecting the NP-ICG-HA in deeper tissues.

### Confirmation of 4T1 Lung Metastasis Mouse Imaging via *Ex Vivo* Analysis of the Tissues

**Figure 6:**
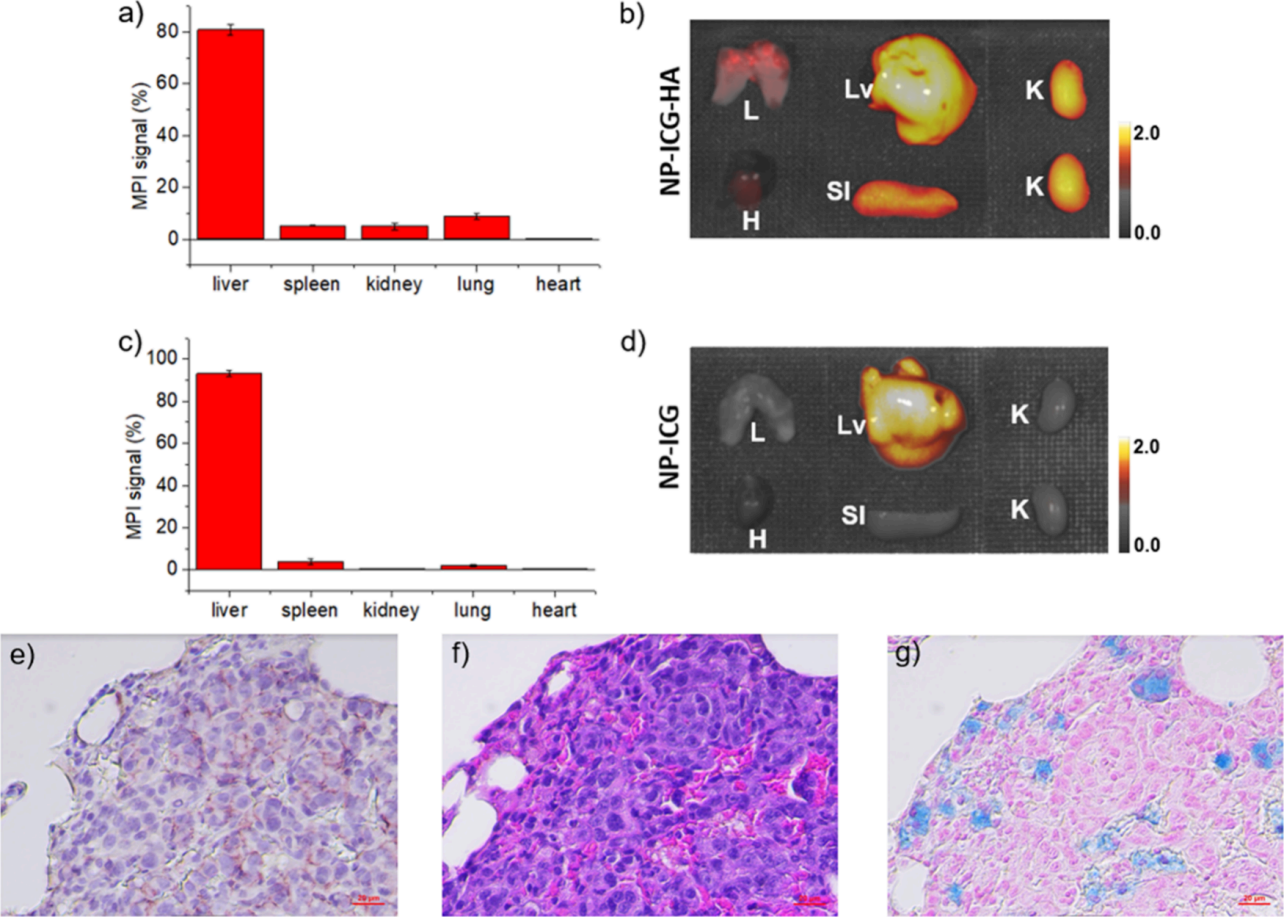
4T1 lung metastasis mice (*n* = 4 for each group) injected with NP-ICG-HA or NP-ICG and then sacrificed at 72 h postinjection. (a) Percentage of MPI signals measured *ex vivo* in main organs and (b) *ex vivo* NIR-FI images of NP-ICG-HA group. (c) Percentage of MPI signals measured *ex vivo* and (d) *ex vivo* NIR-FI images of NP-ICG control group. Lv: liver; K: kidney; Sl: spleen; H: heart; and L: lung. Histological analysis of lung from 4T1 lung metastasis mice receiving NP-ICG-HA. (e) Anti-CD44 IHC stain, (f) H&E stain, and (g) Prussian blue followed by nuclear fast red counterstain. The scale bars are 20 μm.

The tracer administered 4T1 lung metastasis mice were sacrificed at 72 h postinjection, with their organs extracted and imaged by MPI and NIR-FI. The biodistribution of NP-ICG-HA was examined by quantification of MPI (Figure 6a) with the percentage of MPI signal in excised lung determined at 8.8% of the total signals in organs extracted. The *ex vivo* NIR-FI image (Figure 6b) showed a consistent result with MPI with significant intensities in the lungs. In contrast, 4T1 lung metastasis mice receiving NP-ICG only gave significant MPI/NIR-FI signals from excised liver but not from the tumor bearing lungs (Figures 6c and 6d). To further confirm the binding of NP-ICG-HA to metastatic sites in lung, the excised lungs were embedded with paraffin and sliced to 5 μm of thickness for staining. The excised tissues were examined by CD44 immunohistostaining, H&E, and Prussian blue staining (Figures 6e, 6f, and 6g). The brownish area in CD44 IHC slide was co-localized with the dense nucleus stain area in H&E slide, indicating CD44 expression in tumor area. The extensive blue color observed in Prussian blue staining indicated the presence of NP-ICG-HA in metastatic sites in lungs.

## 3 Discussion

Diagnosis of metastasis in addition to primary tumor is crucial to patients with breast cancer since a major cause of breast cancer related death is due to metastasis.^3−5^ While the EPR effects have been often utilized for NP aided cancer diagnosis studies,^16,22,53^ it is less applicable to breast cancer lung metastasis detection due to the small tumor mass in earlier stages of metastasis as compared to the primary tumor.^18−20^ Active targeting can be an attractive approach for the diagnosis of breast cancer lung metastasis.

We report an ICG and HA conjugated IONP (NP-ICG-HA) that enabled active targeting not only to solid tumors but also to metastatic cancer cells in the lungs. The results demonstrated that breast tumor and breast cancer lung metastasis could be detected noninvasively by multimodality, including MPI and NIR-FI. The combination of NP, ICG, and HA was chosen for multiple reasons. First, dextran-coated iron oxide NPs are highly biocompatible and contain functional groups that enable conjugation with targeting molecules, including peptides, polysaccharides, and antibodies. Second, ICG is an FDA approved water-soluble fluorescence dye.^33−39^ Compared to fluorophores excitable only by visible light, ICG enables NIR-FI, which has a better tissue depth penetration. Third, HA is highly biocompatible and can target CD44 overexpressed on breast cancer, including metastatic breast cancer cells.^54−56^ In our study, NP-ICG-HA exhibited no significant toxicity to cells, indicating its good translational potential. Moreover, HA (∼ $200/g) is much less expensive compared to monoclonal antibody (hundreds of $ per 100 μg) as a targeting agent. The NP-ICG-HA showed 2.4-fold stronger signals in MPI compared to those from the commercially available SPION VivoTrax, indicating a better detection sensitivity. For *in vivo* imaging, we demonstrated that NP-ICG-HA detected breast tumor in the MMTV-PyMT model and lung metastasis in the breast cancer lung metastasis model. The *in vivo* results were confirmed by *ex vivo* results and histological studies, showing accumulation of NP-ICG-HA in tumors and metastatic sites in the lungs.

There are limitations to the NP-ICG-HA multimodal imaging platform. Although CD44 is an exciting target and is overexpressed on breast cancer,^26,57^ as the tumor is highly heterogeneous, there may be populations of cancer cells low in CD44 expression, which will escape the detection. For a more comprehensive detection of breast cancer, ligands targeting other biomarkers can be incorporated onto the NP-ICG platform in addition to HA for enhanced selectivity and specificity.

## 4 Conclusions

ICG and HA conjugated SPION were synthesized for imaging of breast cancer at the primary site as well as in the lung. The NP-ICG-HAs integrated the magnetic and optical properties in a single tracer, providing a multimodal imaging platform. We demonstrated that NP-ICG-HAs can bind to 4T1 breast cancer cells through CD44/HA interactions. Moreover, NP-ICG-HAs target CD44 expressing breast tumors in the MMTV-PyMT mouse model, and CD44 expressing breast cancer cells in the lung in 4T1 inoculated BALB/c mice. Thus, NP-ICG-HA is an excellent candidate for breast tumor and lung metastasis imaging.

## 5 Experimental Section

### Materials

Ammonium hydroxide (30% NH_4_OH), 2-chloro-4,6-dimethoxy-1,3,5-triazine (CDMT), dextran (MW: 10 kDa), dimethyl sulfoxide (DMSO), epichlorohydrin, iron(III) chloride hexahydrate (FeCl_3_·6H_2_O), iron(II) chloride tetrahydrate (FeCl_2_·4H_2_O), fetal bovine serum (FBS), formalin solution neutral buffered 10%, *N*-methylmorpholine (NMM), sodium hydroxide (NaOH), Dulbecco’s Modified Eagle Medium (DMEM), Dulbecco’s phosphate-buffered saline (DPBS), RPMI 1640 medium, and penicillin–streptomycin were purchased from Sigma-Aldrich. Sodium hyaluronan (16 kDa) was purchased from Lifecore Biomedicals. CellTiter 96 Aqueous One solution containing 3-(4,5-dimethylthiazol-2-yl)-5-(3-carboxymethoxyphenyl)-2-(4-sulfophenyl)-2*H*-tetrazolium (MTS) was purchased from Promega. The centrifugal filter MWCO (100 kDa) was purchased from EMD Millipore. ICG-NHS ester was purchased from Ruixibiotech.

### Synthesis of NP-NH_2_

FeCl_3_·6H_2_O (1.2 mmol), FeCl_2_·4H_2_O (0.65 mmol), and 4.5 g of dextran (∼10 kDa) were mixed in water (20 mL) and stirred with nitrogen purging the solution for 1 h to remove oxygen from the reaction flask and to improve the magnetic properties of the iron oxide nanoparticles. 30% NH_4_OH solution (0.9 mL) was added in a dropwise manner to the above solution under rapid stirring. The resulting dark greenish solution was heated to 70 °C for 90 min under a nitrogen stream protection to form NPs. The mixture was cooled to room temperature. Ammonium chloride and unreacted dextran were removed by centrifuge through centrifugal filters (MWCO 100 kDa). The colloidal solution of NP in distilled water (25 mL) was mixed with epichlorohydrin (5 mL), and 5 M NaOH (10 mL) then was stirred at room temperature for 24 h to form cross-linked NP. Unreacted epichlorohydrin was removed by centrifuge through centrifugal filters (MWCO 100 kDa). The cross-linked NP was then aminated by adding 30% NH_4_OH solution (10 mL) followed by stirring at 37 °C for 36 h. The excess NH_4_OH in the mixture was removed by centrifuging through centrifugal filters (MWCO 100 kDa) leading to amine-functionalized NP (NP-NH_2_).

### Synthesis of NP-ICG

NP-NH_2_ (4 mg/mL, 3 mL) was mixed with ICG-NHS ester (0.06 mg) in DMSO (1 mL), and the mixture was stirred at 25 °C for 48 h in the dark. The resulting mixture was centrifuged with centrifugal filters (MWCO = 100 kDa) to remove the unreacted ICG-NHS ester.

### Synthesis of NP-ICG-HA

Sodium hyaluronan (∼16 kDa, 100 mg) was dissolved in distilled water (20 mL), and then the Amberlite H^+^ was added to the solution and stirred at 25 °C for 4 h. The resulting solution was filtered and freeze-dried to obtain the protonated HA. HA (40 mg, 0.11 mmol of carboxylic acid), NMM (0.22 mmol), and CDMT (0.08 mmol) were dissolved in a water and acetonitrile mixture (3:2, 6 mL) and stirred at 25 °C for 1 h. NP-ICG (4 mg/mL, 4 mL) was added to the mixture, and the mixture was stirred at 25 °C for 24 h. The unreacted reagents were removed by centrifuging with centrifugal filters (MWCO 100 kDa).

### Characterization of NP-ICG-HA

The hydrodynamic diameter and surface charge of NP-ICG-HA were measured by dynamic light scattering using a Zetasizer Nano zs apparatus (Malvern, U.K.). The morphology of NP-ICG-HA was imaged with an ultrahigh-resolution transmission electron microscope (JEOL 2200FS) operating at 200 kV using Gatan multiscan CCD camera with Digital Micrograph imaging software. The element identification was collected in energy-dispersive X-ray microanalysis (EDX) mode. Absorption and emission of ICG and NP-ICG-HA were measured with a SpectraMax M3 plate reader. The iron concentration of NP-ICG-HA was determined by a Varian 710-ES ICP-OES. The NP-IGC-HA solution was digested with concentrated nitric acid at 60 °C for 2 h and then placed at room temperature overnight. The digested solution was then diluted to a nitric acid concentration of 2% for ICP-OES analysis.

### Competitive ELISA

Competitive ELISA was performed following a literature procedure.^49^ The abilities of NP-ICG-HA and NP-ICG to compete with biotinylated-HA (b-HA) for CD44 binding were measured (*n* = 3 for each group). The 96-well plate was coated with IgG-Fc (3 μg/well) in the wells and then blocked with 5% BSA. The wells were coated with CD44-Fcγ (0.2 μg/well). b-HA (0.5 μg/well), b-HA + NP-ICG (0.5 μg iron/well), and b-HA + NP-ICG-HA (0.5 μg iron/well) were added. Avidin-HRP (1:2000 dilution) was added to all wells, and then chromogenic 3,3′,5,5′-tetramethylbenzidine (TMB) solution (100 μL) was added to each well and incubated for 15 min, or until a blue color appeared. The reactions were then quenched with 0.5 M H_2_SO_4_ (50 μL). Optical absorbance was measured by the SpectraMax M3 plate reader at 450 nm.

### Cell Culture

4T1-Luc2 mouse breast cancer cells were maintained with RPMI 1640 supplemented with 10% FBS and 1% Pen-Strep. RAW 264.7 mouse macrophage cells were maintained with DMEM supplemented with the same materials as described above. The cells were cultured with 5% CO_2_ at 37 °C.

### Biocompatibility of NP-ICG-HA

To evaluate the biocompatibility of NP-ICG-HA, MTS assays were performed (*n* = 3 for each group). RAW 264.7 cells were cultured in a 96-well plate with DMEM containing 10% FBS at 37 °C and 5% CO_2_. The cells were treated with various concentrations of NP-ICG-HA in 100 μL of RPMI-1640 media (0.50, 0.25, and 0.13 mg of Fe/mL) for 24 h at 37 °C and 5% CO_2_ followed by the addition of MTS reagent (20 μL) and then incubated for another 1 h at 37 °C until the brown color developed. The absorption values of the wells were measured at 490 nm with the SpectraMax M3 plate reader.

### Verification of CD44 Expression on 4T1 Cells

To verify the CD44 expression levels on 4T1 cells, 4T1 (5 × 10^5^ cells) was placed in flow cytometry tubes and washed twice with sterile PBS. The cells were then incubated with anti-CD44 APC/Cy7 (IM7, BioLegend catalog no. 103027) in serum-free media (1:200) for 1 h. The cells were washed with sterile PBS three times and then stored on ice until flow cytometry analysis.

### NP-ICG-HA Uptake by CD44 Expressing Cells

To evaluate the binding between NP-ICG-HA and CD44 *in vitro*, 4T1 mouse breast cancer cells were used. Cells were cultured in a Me-tek plate with RPMI-1640 media overnight at 37 °C and 5% CO_2_. The media were removed, and the plates were washed with PBS for three times. The cells were then incubated with NP-ICG-HA or NP-ICG for 1 h at 37 °C followed by three washes with PBS. The cells were fixed with 10% formalin for 15 min and then washed with PBS three times. For confocal analysis, the cells were stained with a DAPI solution (300 nM) for 10 min and then washed with PBS three times. The confocal images were performed with FluoView 1000 LSM (Olympus Corporation). For Prussian blue staining, the cells were incubated with 1:1 mixture of 5% potassium ferrocyanide trihydrate and 10% HCl solution (in PBS) for 1 h at 37 °C and then washed with PBS three times. Images were taken by a Nikon Eclipse Ci microscope with a Nikon DS-Fi3 camera (Nikon Instruments Inc.).

### Mouse Models and Bioluminescence Imaging

All mice were kept in the University Laboratory Animal Resources Facility of Michigan State University. All the experimental procedures and guidelines for animal study were performed under approval of Institutional Animal Care and Use Committee (IACUC) of Michigan State University (Protocol #: 202100095). MMTV-PyMT transgenic mice were purchased from Jackson Laboratory. The female mice spontaneously developed palpable breast cancer in 4 months. BALB/c mice were purchased from Charles River Laboratories. To build a breast cancer lung metastasis model, 4T1-Luc2 cells (5 × 10^5^) were injected through the tail vein. The 4T1-Luc2 cells accumulated in the lungs. To confirm the cell distribution in the mouse, BLI was acquired right after cell injection. d-Luciferin (150 mg/kg) was injected abdominally into the mice 15 min before imaging with IVIS (PerkinElmer).

### Multimodality Imaging

MPI images were acquired on a MOMENTUM MPI scanner (Magnetic Insight Inc.). MPI scanning was performed with the following imaging parameters: (1) scan type: 2D scan; scan mode: Standard; Z FOV: 10.0 cm; 5.7 T/m gradient; (2) the scan type: 3D scan; scan mode: Standard; Z FOV: 10.0 cm; number of projections: 21; 5.7 T/m gradient. CT scan images were acquired on a Micro CT system (PerkinElmer) with a speed scan mode (voltage: 90 kV). 3D MPI/CT data reconstruction and co-registration were processed by VivoQuant (Invicro). The MPI signals from the tumor were integrated through the 3D ROI tool feature in VivoQuant. The percentages of MPI signal from tumor were calculated with the formula (tumor signal/total signal) × 100%. NIR-FI images were acquired on a Trilogy Pearl system (LI-COR Biosciences; exposure time: 500 ms; excitation: 785 nm; signal detection: 820 nm). The fluorescence signals were integrated through NIR-FI images by ImageJ.

### Histological Analysis

Dissected lungs and tumors were fixed in 10% neutral buffered formalin and then processed and vacuum infiltrated with paraffin on the Sakura VIP 2000 tissue processor followed by embedding. Paraffin blocks were sectioned at 5 μm. Hematoxylin and eosin slides were stained on a Leica Autostainer XL. Slides were stained for Prussian blue to detect the ferric form of iron. For CD44 IHC staining, slides were blocked for nonspecific binding with Rodent Block M for 20 min, followed by polyclonal rabbit anti-CD44 antibody dilutions (1:200) and incubations for 1 h at room temperature. Slides were then incubated with rabbit on rodent HRP micro polymer for 20 min with reaction developed utilizing Romulin AEC chromogen for 5 min. Slides were counterstained with CAT hematoxylin in a 1:5 ratio for 1 min. Slides were analyzed with a Nikon Eclipse Ci microscope with a Nikon DS-Fi3 camera (Nikon Instruments Inc.).

## Supporting Information Available

DLS and zeta potential results; additional TEM images, EDX results; magnetic hysteresis curves; competitive ELISA results and method details; MTS assay results; *in vitro* Prussian blue staining images; CD44 IHC staining; NP-ICG-HA and free HA mixture administrated group results; NP-ICG-HA administrated normal BALB/c group *in vivo* results; fluorescence imaging of lung metastasis mice (PDF)

## References

[ref1] Sung H.; Ferlay J.; Siegel R. L.; Laversanne M.; Soerjomataram I.; Jemal A.; Bray F. Global Cancer Statistics 2020: GLOBOCAN Estimates of Incidence and Mortality Worldwide for 36 Cancers in 185 Countries. CA Cancer J. Clin. 2021, 71, 209 10.3322/caac.21660. 33538338

[ref2] Siegel R. L.; Miller K. D.; Wagle N. S.; Jemal A. Cancer statistics, 2023. CA Cancer J. Clin. 2023, 73, 17 10.3322/caac.21763. 36633525

[ref3] Minn A. J.; Gupta G. P.; Siegel P. M.; Bos P. D.; Shu W.; Giri D. D.; Viale A.; Olshen A. B.; Gerald W. L.; Massague J. Genes that mediate breast cancer metastasis to lung. Nature 2005, 436, 518 10.1038/nature03799. 16049480 PMC1283098

[ref4] Jin L.; Han B.; Siegel E.; Cui Y.; Giuliano A.; Cui X. Breast cancer lung metastasis: Molecular biology and therapeutic implications. Cancer. Biol. Ther. 2018, 19, 858 10.1080/15384047.2018.1456599. 29580128 PMC6300341

[ref5] Yousefi M.; Nosrati R.; Salmaninejad A.; Dehghani S.; Shahryari A.; Saberi A. Organ-specific metastasis of breast cancer: molecular and cellular mechanisms underlying lung metastasis. Cell. Oncol. (Dordr.) 2018, 41, 123 10.1007/s13402-018-0376-6. 29568985 PMC12995240

[ref6] Kennel S. J.; Davis I. A.; Branning J.; Pan H.; Kabalka G. W.; Paulus M. J. High resolution computed tomography and MRI for monitoring lung tumor growth in mice undergoing radioimmunotherapy: correlation with histology. Med. Phys. 2000, 27, 1101 10.1118/1.598974. 10841415

[ref7] Gambhir S. S. Molecular imaging of cancer with positron emission tomography. Nat. Rev. Cancer 2002, 2, 683 10.1038/nrc882. 12209157

[ref8] Harris R. S.; Schuster D. P. Visualizing lung function with positron emission tomography. J. Appl. Physiol. 2007, 102, 448 10.1152/japplphysiol.00763.2006. 17038490

[ref9] Almeida S. F. F.; Fonseca A.; Sereno J.; Ferreira H. R. S.; Lapo-Pais M.; Martins-Marques T.; Rodrigues T.; Oliveira R. C.; Miranda C.; Almeida L. P.; Girão H.; Falcão A.; Abrunhosa A. J.; Gomes C. M. Osteosarcoma-Derived Exosomes as Potential PET Imaging Nanocarriers for Lung Metastasis. Small 2022, 18, 2203999 10.1002/smll.202203999. 36316233

[ref10] Branca R. T.; Cleveland Z. I.; Fubara B.; Kumar C. S.; Maronpot R. R.; Leuschner C.; Warren W. S.; Driehuys B. Molecular MRI for sensitive and specific detection of lung metastases. Proc. Natl. Acad. Sci. U. S. A 2010, 107, 3693 10.1073/pnas.1000386107. 20142483 PMC2840507

[ref11] Makela A. V.; Foster P. J. Imaging macrophage distribution and density in mammary tumors and lung metastases using fluorine-19 MRI cell tracking. Magn. Reson. Med. 2018, 80, 1138 10.1002/mrm.27081. 29327789

[ref12] Sato K.; Nagaya T.; Mitsunaga M.; Choyke P. L.; Kobayashi H. Near infrared photoimmunotherapy for lung metastases. Cancer Lett. 2015, 365, 112 10.1016/j.canlet.2015.05.018. 26021765 PMC4508660

[ref13] Cao Q.; Cai W.; Niu G.; He L.; Chen X. Multimodality imaging of IL-18--binding protein-Fc therapy of experimental lung metastasis. Clin. Cancer Res. 2008, 14, 6137 10.1158/1078-0432.CCR-08-0049. 18829492

[ref14] Hong H.; Zhang Y.; Severin G. W.; Yang Y.; Engle J. W.; Niu G.; Nickles R. J.; Chen X.; Leigh B. R.; Barnhart T. E.; Cai W. Multimodality imaging of breast cancer experimental lung metastasis with bioluminescence and a monoclonal antibody dual-labeled with 89Zr and IRDye 800CW. Mol. Pharmaceutics 2012, 9, 2339 10.1021/mp300277f. PMC3500677 22784250

[ref15] Nievelstein R. A. J.; Quarles van Ufford H. M. E.; Kwee T. C.; Bierings M. B.; Ludwig I.; Beek F. J. A.; de Klerk J. M. H.; Mali W. P. T. M.; de Bruin P. W.; Geleijns J. Radiation exposure and mortality risk from CT and PET imaging of patients with malignant lymphoma. Eur. Radiol. 2012, 22, 1946 10.1007/s00330-012-2447-9. 22538627 PMC3411290

[ref16] Yang C.-W.; Liu K.; Yao C.-Y.; Li B.; Juhong A.; Qiu Z.; Huang X. Indocyanine Green-Conjugated Superparamagnetic Iron Oxide Nanoworm for Multimodality Breast Cancer Imaging. ACS Appl. Nano Mater. 2022, 5, 18912 10.1021/acsanm.2c04687. 37635916 PMC10448907

[ref17] Juhong A.; Li B.; Yao C. Y.; Yang C. W.; Liu K.; Agnew D. W.; Lei Y. L.; Luker G. D.; Bumpers H.; Huang X.; Piyawattanametha W.; Qiu Z. Cost-Effective Near Infrared Fluorescence Wide-Field Camera for Breast Tumor Imaging. IEEE Photonics Technol. Lett. 2023, 35, 813 10.1109/LPT.2023.3275470.

[ref18] Maeda H.; Wu J.; Sawa T.; Matsumura Y.; Hori K. Tumor vascular permeability and the EPR effect in macromolecular therapeutics: a review. J. Controlled Release 2000, 65, 271 10.1016/S0168-3659(99)00248-5. 10699287

[ref19] Kobayashi H.; Watanabe R.; Choyke P. L. Improving conventional enhanced permeability and retention (EPR) effects; what is the appropriate target?. Theranostics 2014, 4, 81 10.7150/thno.7193. PMC3881228 24396516

[ref20] Maeda H. Toward a full understanding of the EPR effect in primary and metastatic tumors as well as issues related to its heterogeneity. Adv. Drug Delivery Rev. 2015, 91, 3 10.1016/j.addr.2015.01.002. 25579058

[ref21] Rosenblum D.; Joshi N.; Tao W.; Karp J. M.; Peer D. Progress and challenges towards targeted delivery of cancer therapeutics. Nat. Commun. 2018, 9, 1410 10.1038/s41467-018-03705-y. 29650952 PMC5897557

[ref22] Subhan M. A.; Yalamarty S. S. K.; Filipczak N.; Parveen F.; Torchilin V. P. Recent Advances in Tumor Targeting via EPR Effect for Cancer Treatment. J. Pers. Med. 2021, 11, 571 10.3390/jpm11060571. 34207137 PMC8234032

[ref23] Chen C.; Zhao S.; Karnad A.; Freeman J. W. The biology and role of CD44 in cancer progression: therapeutic implications. J. Hematol. Oncol. 2018, 11, 64 10.1186/s13045-018-0605-5. 29747682 PMC5946470

[ref24] Xu H.; Niu M.; Yuan X.; Wu K.; Liu A. CD44 as a tumor biomarker and therapeutic target. Exp. Hematol. Oncol. 2020, 9, 36 10.1186/s40164-020-00192-0. 33303029 PMC7727191

[ref25] Vadhan A.; Hou M.-F.; Vijayaraghavan P.; Wu Y.-C.; Hu S. C.-S.; Wang Y.-M.; Cheng T.-L.; Wang Y.-Y.; Yuan S.-S. F. CD44 Promotes Breast Cancer Metastasis through AKT-Mediated Downregulation of Nuclear FOXA2. Biomedicines 2022, 10, 2488 10.3390/biomedicines10102488. 36289750 PMC9599046

[ref26] Senbanjo L. T.; Chellaiah M. A. CD44: A Multifunctional Cell Surface Adhesion Receptor Is a Regulator of Progression and Metastasis of Cancer Cells. Front. Cell Dev. Biol. 2017, 5, 18 10.3389/fcell.2017.00018. 28326306 PMC5339222

[ref27] Lesley J.; Hascall V. C.; Tammi M.; Hyman R. Hyaluronan Binding by Cell Surface CD44. J. Biol. Chem. 2000, 275, 26967 10.1016/S0021-9258(19)61467-5. 10871609

[ref28] Banerji S.; Wright A. J.; Noble M.; Mahoney D. J.; Campbell I. D.; Day A. J.; Jackson D. G. Structures of the Cd44-hyaluronan complex provide insight into a fundamental carbohydrate-protein interaction. Nat. Struct. Mol. Biol. 2007, 14, 234 10.1038/nsmb1201. 17293874

[ref29] Misra S.; Hascall V. C.; Markwald R. R.; Ghatak S. Interactions between Hyaluronan and Its Receptors (CD44, RHAMM) Regulate the Activities of Inflammation and Cancer. Front. Immunol. 2015, 6, 201 10.3389/fimmu.2015.00201. 25999946 PMC4422082

[ref30] Gleich B.; Weizenecker J. Tomographic imaging using the nonlinear response of magnetic particles. Nature 2005, 435, 1214 10.1038/nature03808. 15988521

[ref31] Saritas E. U.; Goodwill P. W.; Croft L. R.; Konkle J. J.; Lu K.; Zheng B.; Conolly S. M. Magnetic particle imaging (MPI) for NMR and MRI researchers. J. Magn. Reson. 2013, 229, 116 10.1016/j.jmr.2012.11.029. 23305842 PMC3602323

[ref32] Yu E. Y.; Bishop M.; Zheng B.; Ferguson R. M.; Khandhar A. P.; Kemp S. J.; Krishnan K. M.; Goodwill P. W.; Conolly S. M. Magnetic Particle Imaging: A Novel in Vivo Imaging Platform for Cancer Detection. Nano Lett. 2017, 17, 1648 10.1021/acs.nanolett.6b04865. 28206771 PMC5724561

[ref33] Desmettre T.; Devoisselle J. M.; Mordon S. Fluorescence Properties and Metabolic Features of Indocyanine Green (ICG) as Related to Angiography. Surv. Ophthalmol 2000, 45, 15 10.1016/S0039-6257(00)00123-5. 10946079

[ref34] Ogawa M.; Kosaka N.; Choyke P. L.; Kobayashi H. In vivo molecular imaging of cancer with a quenching near-infrared fluorescent probe using conjugates of monoclonal antibodies and indocyanine green. Cancer Res. 2009, 69, 1268 10.1158/0008-5472.CAN-08-3116. 19176373 PMC2788996

[ref35] Alander J. T.; Kaartinen I.; Laakso A.; Patila T.; Spillmann T.; Tuchin V. V.; Venermo M.; Valisuo P. A review of indocyanine green fluorescent imaging in surgery. Int. J. Biomed. Imaging 2012, 2012, 940585 10.1155/2012/940585. 22577366 PMC3346977

[ref36] Park H. S.; Kim J.; Cho M. Y.; Lee H.; Nam S. H.; Suh Y. D.; Hong K. S. Convenient and effective ICGylation of magnetic nanoparticles for biomedical applications. Sci. Rep. 2017, 7, 8831 10.1038/s41598-017-09627-x. 28821875 PMC5562755

[ref37] Gareev K. G.; Babikova K. Y.; Postnov V. N.; Naumisheva E. B.; Korolev D. V. Fluorescence imaging of the nanoparticles modified with indocyanine green. J. Phys. Conf. Ser. 2017, 917, 042008 10.1088/1742-6596/917/4/042008.

[ref38] Starosolski Z.; Bhavane R.; Ghaghada K. B.; Vasudevan S. A.; Kaay A.; Annapragada A. Indocyanine green fluorescence in second near-infrared (NIR-II) window. PLoS One 2017, 12, e0187563 10.1371/journal.pone.0187563. 29121078 PMC5679521

[ref39] Carr J. A.; Franke D.; Caram J. R.; Perkinson C. F.; Saif M.; Askoxylakis V.; Datta M.; Fukumura D.; Jain R. K.; Bawendi M. G.; Bruns O. T. Shortwave infrared fluorescence imaging with the clinically approved near-infrared dye indocyanine green. Proc. Natl. Acad. Sci. U. S. A 2018, 115, 4465 10.1073/pnas.1718917115. 29626132 PMC5924901

[ref40] Sun X.; Xu Y.; Guo Q.; Wang N.; Wu B.; Zhu C.; Zhao W.; Qiang W.; Zheng M. A Novel Nanoprobe for Targeted Imaging and Photothermal/Photodynamic Therapy of Lung Cancer. Langmuir 2022, 38, 1360 10.1021/acs.langmuir.1c02434. 35060743

[ref41] Cai W.; Gao H.; Chu C.; Wang X.; Wang J.; Zhang P.; Lin G.; Li W.; Liu G.; Chen X. Engineering Phototheranostic Nanoscale Metal–Organic Frameworks for Multimodal Imaging-Guided Cancer Therapy. ACS Appl. Mater. Interfaces 2017, 9, 2040 10.1021/acsami.6b11579. 28032505

[ref42] Tassa C.; Shaw S. Y.; Weissleder R. Dextran-Coated Iron Oxide Nanoparticles: A Versatile Platform for Targeted Molecular Imaging, Molecular Diagnostics, and Therapy. Acc. Chem. Res. 2011, 44, 842 10.1021/ar200084x. 21661727 PMC3182289

[ref43] El-Dakdouki M. H.; Zhu D. C.; El-Boubbou K.; Kamat M.; Chen J.; Li W.; Huang X. Development of multifunctional hyaluronan-coated nanoparticles for imaging and drug delivery to cancer cells. Biomacromolecules 2012, 13, 1144 10.1021/bm300046h. 22372739 PMC5475368

[ref44] El-Dakdouki M. H.; El-Boubbou K.; Kamat M.; Huang R.; Abela G. S.; Kiupel M.; Zhu D. C.; Huang X. CD44 targeting magnetic glyconanoparticles for atherosclerotic plaque imaging. Pharm. Res. 2014, 31, 1426 10.1007/s11095-013-1021-8. 23568520 PMC3823634

[ref45] Hossaini Nasr S.; Tonson A.; El-Dakdouki M. H.; Zhu D. C.; Agnew D.; Wiseman R.; Qian C.; Huang X. Effects of Nanoprobe Morphology on Cellular Binding and Inflammatory Responses: Hyaluronan-Conjugated Magnetic Nanoworms for Magnetic Resonance Imaging of Atherosclerotic Plaques. ACS Appl. Mater. Interfaces 2018, 10, 11495 10.1021/acsami.7b19708. 29558108 PMC5995107

[ref46] Sarkar S.; Kanchibotla B.; Nelson J. D.; Edwards J. D.; Anderson J.; Tepper G. C.; Bandyopadhyay S. Giant increase in the metal-enhanced fluorescence of organic molecules in nanoporous alumina templates and large molecule-specific red/blue-shift of the fluorescence peak. Nano Lett. 2014, 14, 5973 10.1021/nl502990h. 25233371

[ref47] Skardžiu̅tė L.; Kazlauskas K.; Dodonova J.; Bucevičius J.; Tumkevičius S.; Juršėnas S. Optical study of the formation of pyrrolo[2,3-d]pyrimidine-based fluorescent nanoaggregates. Tetrahedron 2013, 69, 9566 10.1016/j.tet.2013.09.044.

[ref48] Zhao Z.; He B.; Nie H.; Chen B.; Lu P.; Qin A.; Tang B. Z. Stereoselective synthesis of folded luminogens with arene-arene stacking interactions and aggregation-enhanced emission. Chem. Commun. 2014, 50, 1131 10.1039/C3CC47696K. 24322508

[ref49] Kamat M.; El-Boubbou K.; Zhu D. C.; Lansdell T.; Lu X.; Li W.; Huang X. Hyaluronic Acid Immobilized Magnetic Nanoparticles for Active Targeting and Imaging of Macrophages. Bioconjugate Chem. 2010, 21, 2128 10.1021/bc100354m. 20977242

[ref50] Yang X.; Sarvestani S. K.; Moeinzadeh S.; He X.; Jabbari E. Effect of CD44 Binding Peptide Conjugated to an Engineered Inert Matrix on Maintenance of Breast Cancer Stem Cells and Tumorsphere Formation. PLoS One 2013, 8, e59147 10.1371/journal.pone.0059147. 23527117 PMC3601067

[ref51] Maglione J. E.; Moghanaki D.; Young L. J. T.; Manner C. K.; Ellies L. G.; Joseph S. O.; Nicholson B.; Cardiff R. D.; MacLeod C. L. Transgenic Polyoma Middle-T Mice Model Premalignant Mammary Disease1. Cancer Res. 2001, 61, 8298. 11719463

[ref52] Lin E. Y.; Jones J. G.; Li P.; Zhu L.; Whitney K. D.; Muller W. J.; Pollard J. W. Progression to Malignancy in the Polyoma Middle T Oncoprotein Mouse Breast Cancer Model Provides a Reliable Model for Human Diseases. Am. J. Pathol. 2003, 163, 2113 10.1016/S0002-9440(10)63568-7. 14578209 PMC1892434

[ref53] Song G.; Zheng X.; Wang Y.; Xia X.; Chu S.; Rao J. A Magneto-Optical Nanoplatform for Multimodality Imaging of Tumors in Mice. ACS Nano 2019, 13, 7750 10.1021/acsnano.9b01436. 31244043

[ref54] Luo Z.; Dai Y.; Gao H. Development and application of hyaluronic acid in tumor targeting drug delivery. Acta Pharm. Sin. B 2019, 9, 1099 10.1016/j.apsb.2019.06.004. 31867159 PMC6900560

[ref55] Wang X.; Song Y.; Yu L.; Xue X.; Pang M.; Li Y.; Luo X.; Hua Z.; Lu C.; Lu A.; Liu Y. Co-Delivery of Hesperetin and Cisplatin via Hyaluronic Acid-Modified Liposome for Targeted Inhibition of Aggression and Metastasis of Triple-Negative Breast Cancer. ACS Appl. Mater. Interfaces 2023, 15, 34360 10.1021/acsami.3c03233. 37432741

[ref56] Michalczyk M.; Humeniuk E.; Adamczuk G.; Korga-Plewko A. Hyaluronic Acid as a Modern Approach in Anticancer Therapy-Review. Int. J. Mol. Sci. 2023, 24, 103 10.3390/ijms24010103. PMC9820514 36613567

[ref57] Al-Othman N.; Alhendi A.; Ihbaisha M.; Barahmeh M.; Alqaraleh M.; Al-Momany B. Z. Role of CD44 in breast cancer. Breast Dis. 2020, 39, 1 10.3233/BD-190409. 31839599

