# Supplementary material for: Active Targeting Hyaluronan Conjugated Nanoprobe for Magnetic Particle Imaging and Near-Infrared Fluorescence Imaging of Breast Cancer and Lung Metastasis
Source: ACS Appl Mater Interfaces. 2024 May 17;16(21):27055–64. doi: 10.1021/acsami.4c01623 (PMC11145589; doi:10.1021/acsami.4c01623)
Supplement: Supplementary file 1 — am4c01623_si_001.pdf [file am4c01623_si_001.pdf]

Supporting Information

Active Targeting Hyaluronan Conjugated Nanoprobe for Magnetic Particle Imaging and Near-infrared Fluorescence Imaging of Breast Cancer and Lung Metastasis

Chia-Wei Yang,[a,b] Kunli Liu,[a,b] Cheng-You Yao,[b,c] Bo Li,[b,c] Aniwat Juhong,[b,c] A. K. M. Atique Ullah,[a,b] Harvey Bumpers,[d] Zhen Qiu,[b,c,e] Xuefei Huang[a,b,e]*

[a] Department of Chemistry, Michigan State University, East Lansing, MI, 48824 USA
[b] Institute for Quantitative Health Science and Engineering, Michigan State University, East Lansing, MI, 48824 USA
[c] Department of Electrical and Computer Engineering, Michigan State University, East Lansing, MI, 48824 USA
[d] Department of Surgery, Michigan State University, East Lansing, MI, 48824 USA
[e] Department of Biomedical Engineering, Michigan State University, East Lansing, MI, 48824 USA

Email: huangxu2@msu.edu

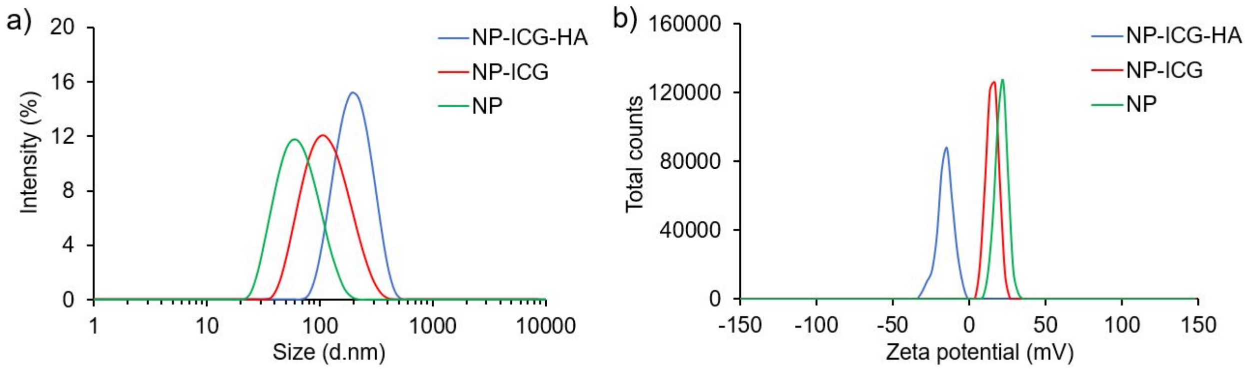

Figure S1. Characterizations of NP, NP-ICG, and NP-ICG-HA (a) hydrodynamic diameter and (b) Zeta potential. NP, NP-ICG, and NP-ICG-HA were diluted to Fe concentration of 0.05 mg/mL in PBS for these measurements.

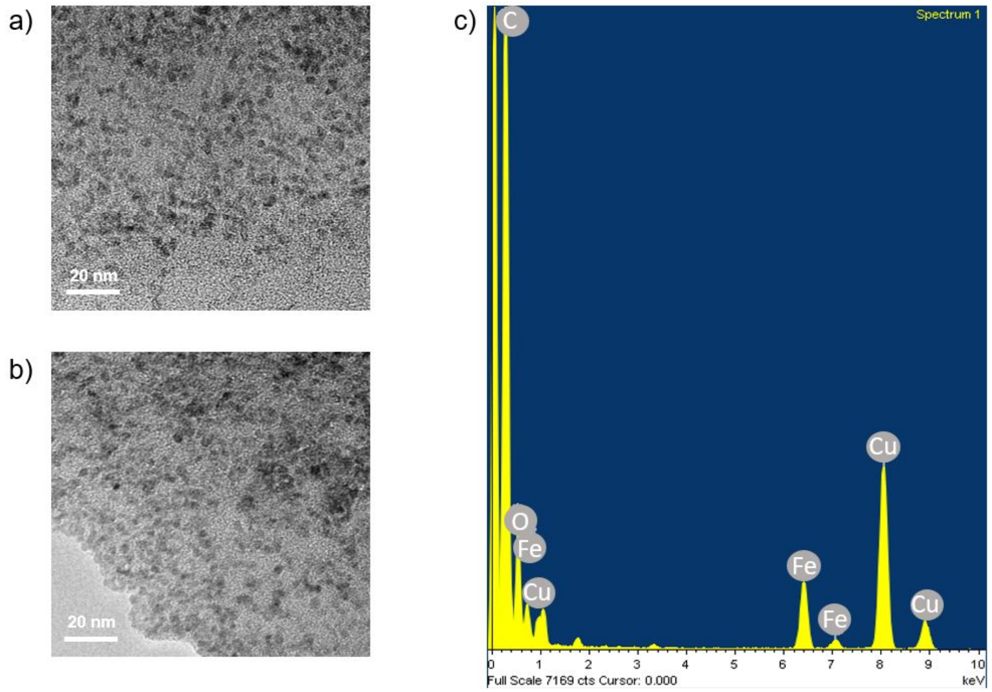

Figure S2. The TEM images of (a) NP and (b) NP-ICG. (c) Energy-dispersive X-ray spectroscopy of NP-ICG-HA. The copper element was from the copper grid used for the measurement.

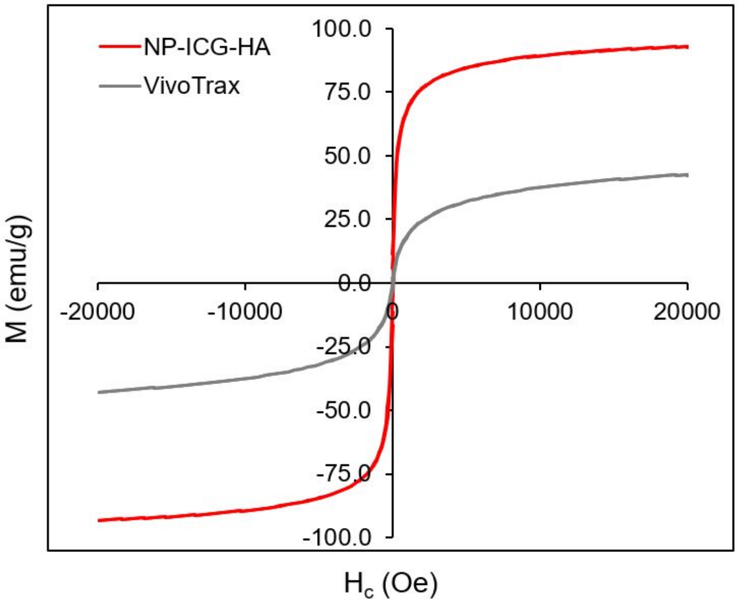

Figure S3. The magnetic hysteresis curves of NP-ICG-HA and VivoTrax at 300K.

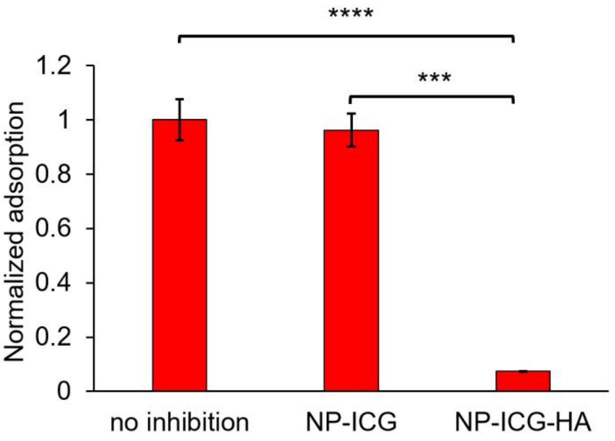

Figure S4. Competitive ELISA experiment showed that NP-ICG-HA competed with biotinylated-HA (b-HA) for CD44 binding while the corresponding NP-ICG did not (n=3). ELISA wells were coated with CD44. b-HA was added to the wells (no inhibition column), which bound with immobilized CD44. Upon removal of unbound ligand, streptavidin-HRP conjugate was added, which allowed the semi-quantification of the amount of bound b-HA based on the absorbance of the wells. For competitive ELISA, NP-ICG or NP-ICG-HA was added together with b-HA to CD44 coated wells (NP-ICG and NP-ICG-HA columns respectively). NP-ICG-HA could bind with CD44 reducing the amount of b-HA retained in the wells by CD44, thus significantly reducing the absorbance values of the wells. Statistical analysis was performed through one-way ANOVA analysis. ***, $p < 0.001$; ****, $p < 0.0001$.

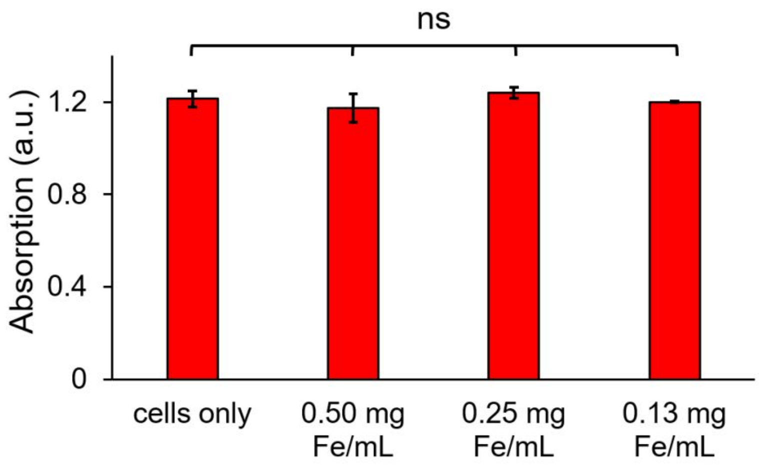

Figure S5. Cell viability test by the MTS assay. RAW 264.7 cells were incubated with various concentrations of NP-ICG-HA for 24 h at 37°C (n=3). The absorption values at 490 nm were acquired as a measure of the number of live cells. At the concentrations evaluated, the NP-ICG-HA did not significantly impact the cell viability. Statistical analysis was performed through one-way ANOVA analysis. ns, non-significant.

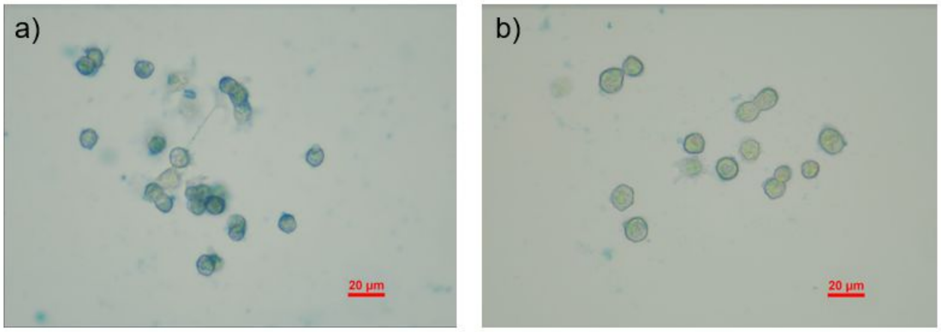

Figure S6. 4T1 cells were incubated with (a) NP-ICG-HA and (b) NP-ICG for 4 h at 37°C then stained with Prussian Blue, after being washed with PBS for three times following each staining. The significantly stronger blue color in panel a than that in panel b suggests the higher uptake of NP-ICG-HA as compared to NP-ICG. Scale bars are 20 µm.

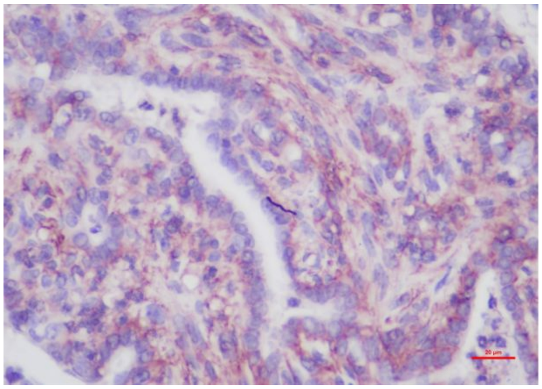

Figure S7. Excised breast tumor from MMTV-PyMT. Tumor slides were performed with CD44 IHC staining (brown color). The scale bar is 20 μm.

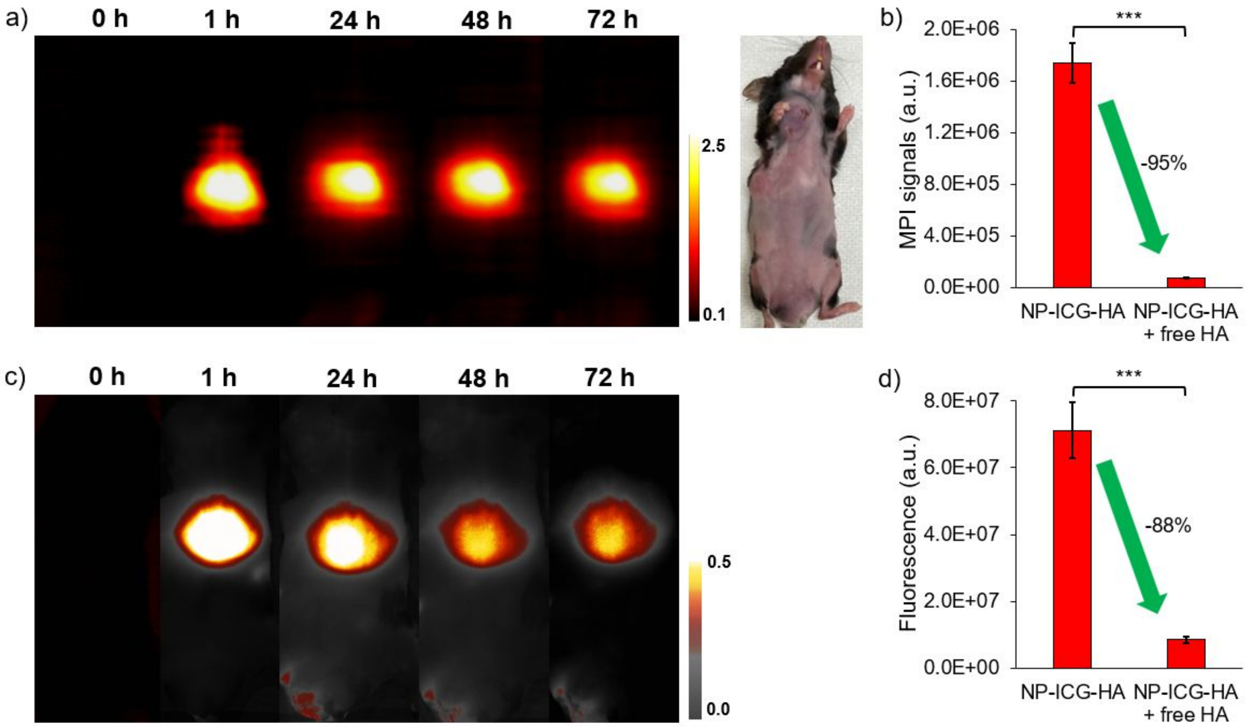

Figure S8. MMTV-PyMT mice were injected with NP-ICG-HA and free HA mixture (n=3). (a) 2D MPI images at coordinated time points. (b) Integration of MPI signals in tumors area at 24 h post-injection showed a 95% reduction of signals. (c) NIR-FI images indicating little NPs in the tumor. (d) Integration of fluorescence in tumors area at 24 h post-injection showed an 88% reduction of signals. Statistical analysis was performed through one-way ANOVA analysis. ***, $p < 0.001$

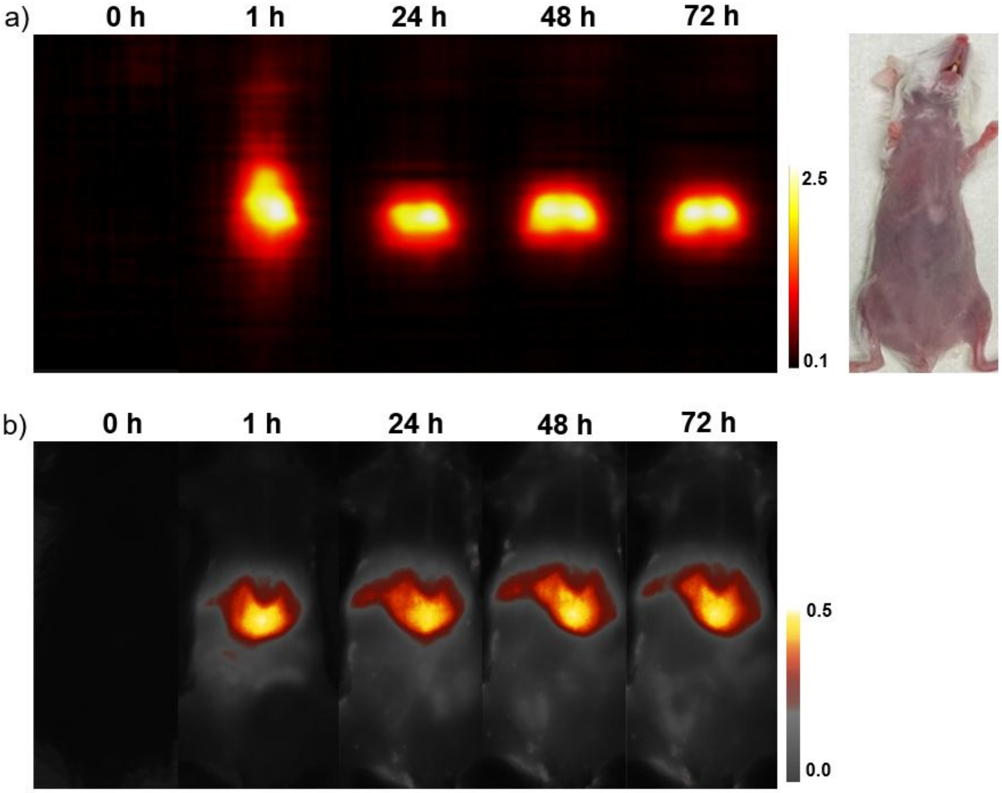

Figure S9. Normal BALB/c (n=3) were administrated with NP-ICG-HA. (a) 2D MPI images (b) fluorescence imaging at coordinated time points showing little NP signals from lung area.

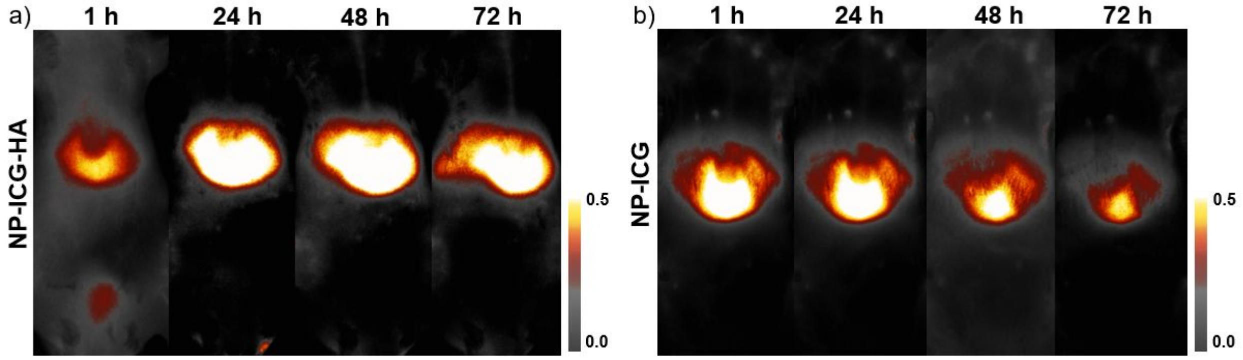

Figure S10. Fluorescence images of (a) lung metastasis mice (n=4 for each group) injected with NP-ICG-HA and (b) NP-ICG at different time points.
